# Satisfying Consumer Preferences: The Packaging Design of Guizhou Red Sour Soup

**DOI:** 10.3390/foods13233806

**Published:** 2024-11-26

**Authors:** Huafeng Quan, Yiting Li, Qin Li, Dashuai Liu

**Affiliations:** 1 College of Big Data and Statistics, Guizhou University of Finance and Economics, Guiyang 550025, China; huafengquan@mail.gufe.edu.cn (H.Q.);; 2 Key Laboratory of Advanced Manufacturing Technology, Ministry of Education, Guizhou University, Guiyang 550025, China; 3 School of Art Design and Media, East China University of Science and Technology, Shanghai 200237, China; liudashuaichn@163.com

**Keywords:** red sour soup, consumer behavior, packaging design, Miao culture, Kansei engineering, semiotics

## Abstract

Red sour soup (RSS) is a traditional food with rich cultural connotations and nutritional value, unique to the Kaili region of Guizhou Province, China. However, the existing packaging design lacks cultural characteristics and visual appeal, which greatly limits its market potential. This study proposes a systematic research framework for RSS packaging design by integrating NLP, Kansei engineering, and semiotics. First, we mined consumers’ dual requirements by analyzing online reviews from e-commerce and tourism platforms using NLP and LDA. Second, we used Kansei engineering to construct a mapping model between consumers’ requirements and design elements. Notably, in the semantic space, we innovatively introduced the concept of a semantic network, considering the relationships between Kansei words for the first time. Finally, we proposed integrating the four dimensions of semiotics into the four stages of design, guiding the application of batik culture in packaging design. Based on this framework, we created a set of RSS packaging designs that integrate modern design concepts and traditional Miao cultural elements. The new design showed significant attractiveness in testing, with 100% of subjects preferring it, fully demonstrating the effectiveness and consumer acceptance of our approach. This study provides new methods and ideas for packaging in the food industry, which has positive significance for the modernized marketing of traditional foods.

## 1. Introduction

Red sour soup (RSS) is a traditional condiment in the Qiandongnan region of Guizhou Province, particularly popular among the Miao people in Kaili [1]. Guizhou’s long history of consuming sour foods can be attributed to its humid climate, which promotes natural fermentation, and the historical scarcity and high cost of salt due to the region’s lack of salt mines, as well as the feudal dynasties’ control over salt supplies. As a result, “replacing salt with acid” became a survival strategy for the people of Guizhou, as sour foods can help reduce the loss of sodium ions [2].

RSS is a semi-solid or liquid food with a bright red color and sour taste. It is primarily made from fresh tomatoes and red peppers, supplemented with ginger, salt, and white wine, and produced through a fermentation process. The bright red color is derived from the tomatoes and peppers. In Miao culture, red symbolizes passion and happiness. The bright red color also influences consumers’ visual and gustatory perceptions. RSS not only has a history spanning thousands of years, but has also received many honors [3]. For example, in 2013, it was listed as a national geographical indication protected product. In 2021, Kaili sour soup fish was even listed as the fifth batch of national intangible cultural heritage.

**Figure 1:**
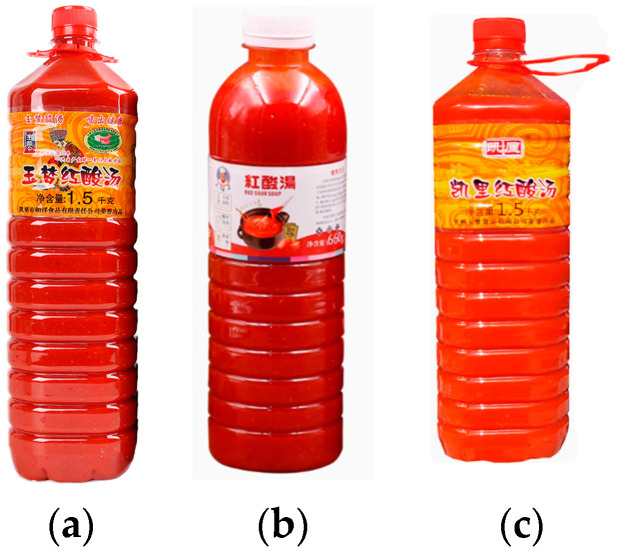
The current packaging design of red sour soup (RSS) in Guizhou. (**a**) The Yumeng brand; (**b**) the Liuhuzi brand; (**c**) the Kaili brand.

Recent academic research on RSS has focused on microbial communities, fermentation optimization, nutritional components, volatile flavor substances, and functional properties [4,5,6,7]. However, despite its health benefits, RSS faces challenges in market promotion and dissemination, with packaging being a key factor. As shown in Figure 1, the current crude packaging, often consisting of transparent plastic bottles, fails to showcase the product’s characteristics and cultural value, leading to decreased consumer satisfaction. This issue is not unique to RSS and is common among most local specialty foods.

To improve the attractiveness of local specialty foods, researchers suggest integrating cultural elements such as traditional patterns, colors, and craftsmanship with modern design concepts [8]. Table 1 summarizes the research on the application of culture in food packaging.

The analysis reveals that current food packaging designs often face issues such as a lack of design awareness, neglect of consumer needs, and poor cultural adaptability. Although studies have proposed solutions, they remain at the macro level and lack the support of actual design cases and modern design methods.

In terms of design awareness, current designs often overlook the uniqueness and market positioning of the food product, resulting in a lack of differentiated competitive advantage. Regarding consumer needs analysis, traditional methods such as face-to-face surveys, focus groups, and in-depth interviews, although capable of obtaining insights, are limited in data scale. Recent studies have focused on large-scale textual data. Tian et al. [13] manually analyzed consumer needs based on Yelp reviews, while Heng et al. [14] employed Latent Dirichlet Allocation (LDA) for automatic topic extraction. However, they only utilize data from a single e-commerce platform, potentially introducing bias in the analysis of consumer needs. Furthermore, these studies fail to further transform the identified needs into design elements.

Regarding the transforming of consumer needs, Kansei engineering can convert user requirements into specific design elements by constructing a mapping model. For instance, Effendi et al. [15] and Maleki et al. [16] employed this method to analyze the optimal designs for mint hard candy and chocolate packaging. However, they treated Kansei words as independent entities, without considering their relationships and roles in the overall structure, which may result in an incomplete selection of Kansei words.

Regarding the adaptability of culture, the integration of culture and packaging presents certain difficulties. This is not a simple matter of putting cultural elements onto the packaging; rather, it requires the in-depth consideration of several issues: how to transform abstract cultural connotations into concrete visual symbols, how to strike a balance between traditional and modern aesthetics, and how to accurately convey the intended design meaning across different cultural contexts. Semiotics provides a valuable perspective for understanding cultural symbols, but studies applying semiotics to design practice are rare, and few combine it with Kansei engineering.

In RSS packaging design, there is an urgent need for a novel research framework that integrates consumer requirements, packaging elements, and cultural analysis to form a systematic guiding method. From this perspective, this study attempts to contribute new ideas by combining consumer requirements with Miao culture to enhance the attractiveness and cultural connotations of RSS packaging, stimulating consumers’ purchase intention and brand loyalty. The main contributions of this paper are as follows:

1. Multi-source data fusion for consumer requirement acquisition, utilizing user online reviews from e-commerce and tourism platforms to provide comprehensive and objective insights.
2. The improvement and application of Kansei engineering, introducing the concept of semantic network to consider the importance and interrelationships of Kansei words, enhancing the depth and accuracy of consumer needs analysis.
3. The integration of semiotic theory and packaging design process, matching the four dimensions of semiotics with the four key links of the design process, thereby providing a theoretical basis and practical guidance for applying traditional cultural elements in modern packaging design.
4. The integration of interdisciplinary research methods, constructing a comprehensive research framework covering the entire process from consumer requirement acquisition to packaging solution design.

The significance of this research is not limited to the improvement of RSS packaging, but also provides a replicable method and idea for the packaging design of other local specialty foods. It helps promote the application of traditional cultural elements in modern food packaging design, enhance the market promotion of local specialty foods, and provide new ways for cultural inheritance and innovation.

The overall structure of this paper is as follows. In Section 2, we reviewed the related works. In Section 3, we presented the research framework in detail, which included LDA, semiotics, and Kansei engineering. In Section 4, we carried out the specific research work, and verified the feasibility of the framework by designing RSS packaging. Section 5 summarizes and discusses the limitations and future directions of the research.

## 2. Related Works

### 2.1. Food Packaging Design

Food packaging design, which accounts for 51% of global packaging usage [17], is crucial for product marketing and consumer decisions. Beyond its basic protective function, modern food packaging now serves multiple roles, including visual appeal, information delivery, and brand identity. Research shows that packaging design directly influences consumers’ purchasing behavior.

In visual design, Velasco et al. [18] found that visual elements like color, shape, and font can shape consumers’ expectations of food taste and quality. For example, round packaging is often associated with sweeter foods, while angular packaging suggests sourness. Sustainability has also become a key consideration in food packaging design [19,20]. Lindh et al. [21] observed that consumers increasingly prefer eco-friendly packaging materials. Khandeparkar et al. [22] highlighted the potential of bioplastics, derived from renewable polymers like corn starch, sugarcane, or algae, as a sustainable and biodegradable packaging solution.

The application of cultural elements in food packaging is receiving increasing attention. Zhao [9] based on his research on Henan cultural resources in China, emphasizing the importance of integrating traditional culture into packaging design. Wu and Chen [12] also attributed the success of modern Japanese packaging design to its deep roots in traditional culture. Tang and Liu [23] discussed how to apply cultural elements such as New Year pictures, paper-cutting art, embroidery art, and shadow puppetry in packaging design and how these art forms bring new ideas and added value. Liu [10] discussed the specific embodiment of culture in packaging design, including pattern, color, and font. Underwood [24] pointed out that food packaging is an important carrier for building cultural connotations and brands, and the related cultural symbols should be mined and transformed. In addition, Huang’s [25] findings prove that successful cultural packaging design can even improve consumers’ mental health and alleviate obsessive–compulsive disorder. These studies only provide some discussions at a macro level, and rarely provide specific guidance. Moreover, the integration of culture and packaging should also consider adaptability.

Current research rarely combines Kansei engineering and semiotics in food packaging design, leaving a gap in systematic design methodology. These two approaches are complementary: Kansei engineering can be used to identify the key design elements that influence consumer perception, while semiotics can guide the integration of cultural symbols with design elements, ensuring that the incorporation of cultural elements is not superficial and resonates with consumers on both emotional and cultural levels. This study bridges this gap by integrating Natural Language Processing (NLP), Kansei engineering, and semiotics to develop a framework that transforms Miao batik symbols into modern food packaging design, specifically for RSS packaging.

### 2.2. Kansei Engineering in Design

Kansei, a Japanese term encompassing sensation, emotion, and perception, forms the basis of Kansei engineering. Developed by Nagamachi in the late 20th century, this engineering approach analyzes the relationship between consumer perception and product design features [26]. It has since been applied across various fields, from automotive to interior design, as well as in consumer behavior research.

The original Kansei engineering also has room for improvement, and researchers have made some efforts in this direction. Kuo et al. [27] used a dual semantic space and multi-sensory experiments to capture users’ multidimensional feelings. Some scholars have explored the introduction of AI to improve design efficiency and visualization [28]. Gan et al. [29] used DCGAN to generated design solutions that conform to consumers’ perceptual preferences, shortening the design cycle and improving innovation. Lai et al. [30] developed a framework integrating various AI techniques to mine more accurate requirements and later proposed a behavior-enhanced BERT model [31].

Kansei engineering focuses on the acquisition and measurement of Kansei. Researchers usually collect Kansei words through various methods and screen them using techniques like word frequency, TF-IDF, clustering, and factor analysis. However, these methods are limited by participants’ experience, which may lead to inaccurate results. To overcome this, some scholars have focused on textual big data from online shopping platforms. Yang et al. [32] extracted adjectives from online reviews and used factor analysis, while Shieh et al. [33] clustered the collected Kansei words. In our previous research [34], we combined TF-IDF with EPA to propose a new Kansei word screening method.

Kansei measurement is divided into psychological and physiological measurements. Physiological measurement indirectly associates Kansei by recording subjects’ physiological signals, while psychological measures focus on subjects’ words, behaviors, and expressions, using tools like the Semantic Differential (SD) scale and the Likert scale.

Existing Kansei engineering methods have limitations in selecting Kansei words, as they analyze them as separate individuals without fully considering their relationships and role in the overall network structure, potentially leading to a lack of comprehensiveness. To address this, we introduce a semantic network, construct an association network of Kansei words, and filter them by key nodes. This innovation helps capture consumers’ perceptual requirements more comprehensively and provides a new method for applying Kansei engineering in food packaging design, aiming to offer more accurate guidance for RSS packaging design while contributing new research ideas to Kansei engineering.

## 3. Methods

### 3.1. Research Framework

**Figure 2:**
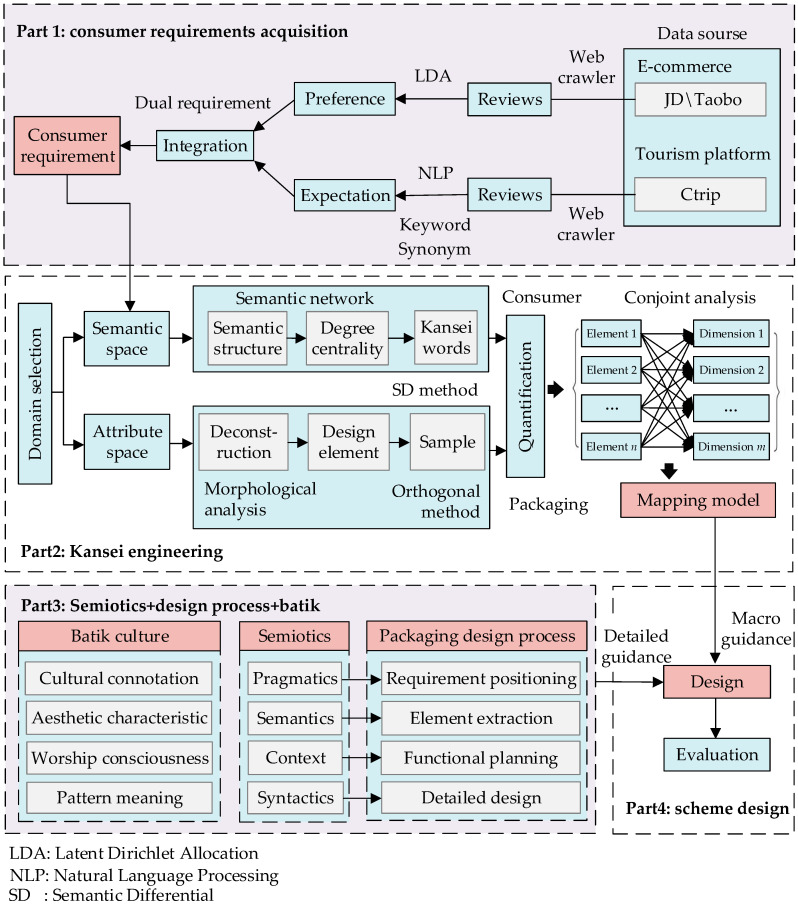
Research framework.

To improve the visual appeal and cultural connotation of RSS packaging, we constructed a research framework that integrates methods such as web crawling, NLP, Kansei engineering, and semiotics. As shown in Figure 2, the framework mainly consists of four parts.

Part 1 focuses on the acquisition of consumer requirements, the core link of the design process, and directly determines the design direction. We extracted reviews from tourism and e-commerce platforms using web crawling, and applied NLP and LDA to identify requirements related to cultural tourism products and RSS packaging, revealing consumer requirements. The criteria for selecting review platforms include (i) relevance to the research objectives, i.e., cultural tourism and food packaging; (ii) the popularity and market share of the platforms; (iii) the availability and richness of user-generated content.

Part 2 involves using Kansei engineering to construct a mapping model between packaging design elements and consumer perceptions. We introduced a semantic network to filter out key Kansei words, used morphological analysis to deconstruct RSS packaging elements, and conducted orthogonal experiments to obtain representative samples. We then evaluated consumers’ perceptual responses using the SD method and established a mapping model through conjoint analysis. This step transforms qualitative perceptual requirements into quantitative design guidance.

Part 3 involves applying semiotics to integrate batik into RSS packaging. We matched the semantics, syntactics, pragmatics, and context in semiotics with consumer requirement positioning, design element extraction, functional design, and detailed design in the design process, respectively. This ensures that cultural elements in the packaging are not merely superficial decoration but convey deep emotion and cultural connotations.

Part 4 focuses on the design and evaluation of the RSS packaging. Guided by the mapping model and semiotics, we designed the packaging scheme for Kaili RSS, synthesizing insights from the previous parts to create a packaging design that is both visually appealing and culturally significant.

### 3.2. LDA

LDA is a widely used probabilistic topic modeling algorithm that discovers hidden semantic structures in a large collection of documents. It is an unsupervised learning method, meaning it does not require the prior labeling or annotation of the documents. The main idea behind LDA is that each document in a corpus is a mixture of various topics, and each topic is characterized by a distribution of words.

**Figure 3:**
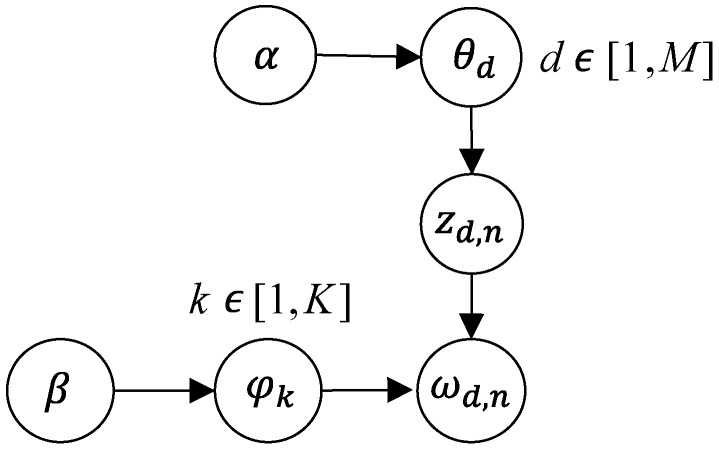
The topic and word generation process of the Latent Dirichlet Allocation (LDA) model.

The goal of LDA is to infer the latent topic structure from the observed documents. It achieves this by iteratively adjusting the topic assignments for each word and updating the topic–word distributions until convergence. The result is a set of topics, each represented by a distribution over words, and a topic distribution for each document in the corpus. The working principle of LDA can be simply summarized as follows: (i) assign a set of topics to each document; (ii) assign a set of words to each topic; (iii) adjust the allocation of topics and words until the most reasonable combination is identified. Figure 3 illustrates the topic and word generation process in LDA.

The generation process of LDA is as follows:

- Generate the topic distribution *θ_d_* for document d from a Dirichlet distribution *α*.
- Generate the topic *z_d,n_* of the nth word in d from *θ_d_*.
- Generate the word *w_d,n_* from the word distribution *φ_z__d,n_* corresponding to topic *z_d,n_*.
- For each topic *k*, generate the word distribution *φ_k_* for topic *k* from a Dirichlet distribution *β*.

Here, *α* and *β* are hyper parameters of the LDA model, controlling the prior shapes of the topic distribution and word distribution, respectively.

Based on the above process, LDA can be represented by the following joint probability distribution:

(1) $$p(W,Z,\theta ,\varphi |\alpha ,\beta )=\prod_{k=1}^{K}p(\varphi_{k}|\beta )\prod_{d=1}^{M}p(\theta_{d}|\alpha )\prod_{n=1}^{N_{d}}p(z_{d,n}|\theta_{d})p(w_{d,n}|\varphi_{z_{d,n}})$$

where *W* represents all words, *Z* represents all topics, *K* is the number of topics, *M* is the number of documents, *N_d_* is the number of words in document *d*, and *θ* and *φ* represent topic distribution and word distribution, respectively.

In practical applications, we usually focus on the posterior distribution *p*(*Z*, *θ*, *φ* |*W*, *α*, *β*); that is, inferring the topic distribution of documents, the word distribution of topics, and the topic of each word given the observed words and hyper parameters. Since this posterior distribution is difficult to solve directly, approximate inference algorithms such as variational inference and Gibbs sampling need to be used.

In this study, we use the variational inference method. Variational inference approximates the true posterior distribution *p*(*Z*, *θ*, *φ* |*W*, *α*, *β*) by introducing a variational distribution *q*(*Z*, *θ*, *φ*) and minimizing the KL divergence between them:

(2) $$\begin{array}{l} \underset{q}{\min}KL(q(Z,\theta ,\varphi )∥p(Z,\theta ,\varphi |W,\alpha ,\beta ))\\ =\underset{q}{\min}(E_{q}[\log q(Z,\theta ,\varphi )]-E_{q}[\log p(Z,\theta ,\varphi |W,\alpha ,\beta )])\\ =\underset{q}{\min}(E_{q}[\log q(Z,\theta ,\varphi )]-E_{q}[\log p(W,Z,\theta ,\varphi |\alpha ,\beta )]+\log p(W|\alpha ,\beta ))\\ =\underset{q}{\min}(-L(q)+\log p(W|\alpha ,\beta ))\\ =\underset{q}{\max}L(q)-\log p(W|\alpha ,\beta ) \end{array}$$

*L*(*q*) is the variational lower bound, defined as:

(3) $$L(q)=E_{q}[\log p(W,Z,\theta ,\varphi |\alpha ,\beta )]-E_{q}[\log q(Z,\theta ,\varphi )]$$

Since log *p*(*W* | *α*, *β*) is a constant term independent of the variational distribution *q*(*Z*, *θ*, *φ*), minimizing the KL divergence is equivalent to maximizing the variational lower bound *L*(*q*). Through variational inference, the parameters of the LDA model can be obtained, and then the topic distribution of documents, the word distribution of topics, and the topic of each word can be estimated.

In practice, it is crucial to predetermine an appropriate number of topics *K*. Setting *K* too low may result in broad topics that lack detail; conversely, setting it too high can result in fragmented topics that are difficult to form effective interpretations [35]. While the perplexity and coherence are useful metrics at this stage, domain knowledge and a manual inspection of the results are also important. In addition, LDA excels at processing large-scale text. Therefore, we are consolidating the individual reviews into a single document. This strategy can help us to extract meaningful topics from short texts like online reviews, which are typically challenging for LDA to process individually.

### 3.3. Semiotics

Semiotics is a theory jointly initiated by Swiss linguist Ferdinand de Saussure and American philosopher Charles Sanders Peirce in the early 20th century, studying the essence of symbols, the laws of symbol development and change, the various meanings of symbols, and the relationship between symbols and human activities [36].

**Figure 4:**
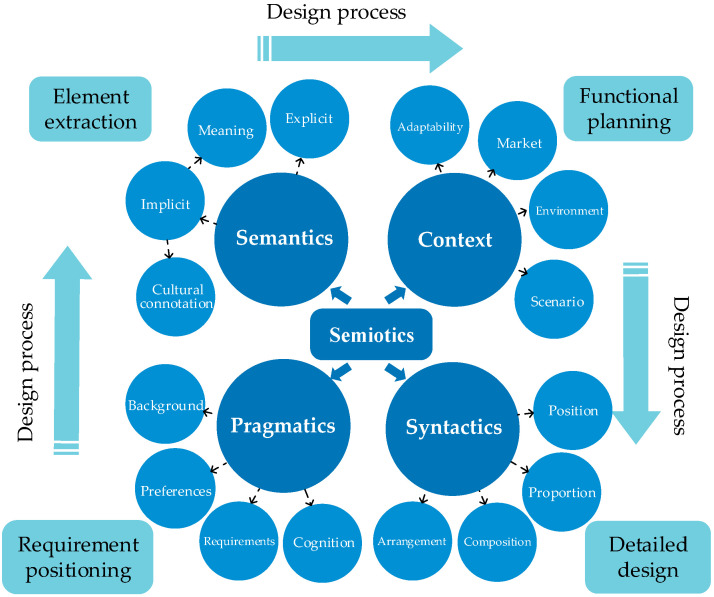
Correspondence between design semiotics and design process.

With the development and integration of semiotics and design, design semiotics was derived, embodying the unity of its signifier (design expression) and that which is signified (design connotation), including four dimensions: semantics, syntactics, pragmatics, and context. We analyzed the correspondence between these dimensions and the packaging design process, as shown in Figure 4.

1. The pragmatic dimension corresponds to consumer requirement positioning, focusing on the relationship between symbols and users, including consumers’ cognition and preference for symbols. For example, in RSS packaging design, we need to consider the acceptance of the target consumer group for batik culture.
2. The semantic dimension corresponds to the extraction of design elements, including explicit semantics (such as shape) and implicit semantics (such as meaning). Appropriate design elements are extracted according to the semantics of symbols. In our research, it involves choosing the appropriate batik pattern based on its cultural connotation and shape.
3. The context dimension corresponds to functional design, considering the adaptability of design in social environments and usage scenarios, such as how RSS packaging stands out in a shopping environment while also conveying traditional culture.
4. The syntactic dimension corresponds to the detailed design stage, focusing on the organization and structure of design elements. In RSS, this involves how to organize text, different batik patterns, and other design elements on the packaging to achieve an aesthetic and functional effect, considering aspects such as balance, harmony, and visual hierarchy.

This integration of semiotics with the packaging design process bridges traditional cultural elements and modern design, ensuring that the packaging not only attracts consumers but also effectively conveys the cultural connotation of the food.

### 3.4. Kansei Engineering

Kansei engineering is a user-centric research method that transforms consumers’ perceptual requirements into product design elements to enhance consumer satisfaction [37,38]. Consumer satisfaction refers to the extent to which a company’s products or services meet consumer expectations. Kansei engineering establishes a quantitative mapping relationship between RSS packaging design elements and consumers’ perceptual requirements through four main steps:

1. Product domain selection: Identifying the specific research object and considering market demand, corporate strategy, and other factors to ensure the research results have practical application value and market relevance. We chose RSS packaging as the research object due to its unique cultural background and market potential.
2. Product semantic space construction: Collecting and selecting consumers’ subjective feelings about the product. These feelings are expressed in the form of adjectives or phrases, called Kansei words. We collected Kansei words from online reviews and used semantic networks to construct an association network between them, identifying key nodes through the degree centrality index. This method not only evaluates the importance of individual Kansei words but also considers the interrelationships among them.
3. Product attribute space construction: In this step, first, morphological analysis is used to deconstruct the product appearance, resulting in a series of independent design elements. Then, the sample set is obtained by combining these elements through orthogonal experiments. Compared to the full-profile method, this approach yields samples that reflect the diversity of the design space while reducing the workload and cost of evaluation.
4. Mapping model construction: Reveal the relationship between semantic and attribute spaces. This study uses the SD method to quantify consumers’ perceptual evaluations of products and constructs a Kansei matrix *A*. In matrix *A*, *a_ij_* represents the average score of the *i*th sample on the *j*th perceptual dimension, where *m* represents the number of samples and *n* represents the number of perceptual dimensions.
  (4) $$A=\{a_{ij}\}=\left[\begin{array}{cccc} a_{11} & a_{12} & \cdots & a_{1n}\\ a_{21} & a_{22} & \cdots & a_{2n}\\ \vdots & \vdots & \ddots & \cdots \\ a_{m1} & a_{m2} & \cdots & a_{mn} \end{array}\right](i=1,2,\cdots ,m,j=1,2,\cdots ,n)$$

To ensure the reliability of the Kansei matrix, this study introduces Cronbach’s *α* coefficient as a measure of internal consistency, which is calculated as follows:

(5) $$\alpha =\frac{n}{n-1}*(1-\frac{\sum_{j=1}^{n}(\frac{1}{m-1}*\sum_{i=1}^{m}{\left(a_{ij}-\frac{1}{m}*\sum_{i=1}^{m}a_{ij}\right)}^{2})}{\frac{1}{m-1}*\sum_{i=1}^{m}(\sum_{j=1}^{n}a_{ij}-\frac{1}{m}*\sum_{i=1}^{m}a_{ij}{)}^{2}})$$

The value range and corresponding internal consistency degree of Cronbach’s *α* coefficient are shown in Table 2.

After the consistency test, conjoint analysis is used to integrate the design elements and Kansei matrix of the samples to construct a mapping model. Conjoint analysis is a quantitative method widely used in product design and consumer preference research. Utility and importance are at the core of conjoint analysis. The utility value quantifies the performance intensity of design elements in each perceptual dimension, while the importance value reveals the contribution degree of each design element to the overall perceptual experience.

The utility value of level *f* in element *e* under perceptual dimension *j* is calculated as follows:

(6) $$u_{ef}\_j=\frac{\sum_{i=1}^{m}a_{ij}*S_{ief}}{n_{ef}\_j}$$

where *n_ef__ j* represents the number of occurrences of level *f* in element *e* under dimension *j*; *S_ief_* is an indicator function, if the level in element *e* of the *i*th sample is *f*, then *S_ief_* = 1, otherwise *S_ief_* = 0.

If the utility values *u_ef__j* of each level *f* in element *e* differ to a very small degree, then its importance is low; conversely, its importance is high. Let the total number of design elements be *E*; then, the importance value of element *e* under dimension *j* is calculated as follows:

(7) $$I_{e}\_j=\frac{\max(u_{ef}\_j)-\min(u_{ef}\_j)}{\sum_{e=1}^{E}\left[\max(u_{ef}\_j)-\min(u_{ef}\_j)\right]}$$

## 4. Case Study

### 4.1. Consumer Semantic Space Construction

#### 4.1.1. Consumer Expectation Acquisition and Preprocessing

Ctrip (https://www.ctrip.com), as China’s leading online travel platform, possesses a vast repository of user review data characterized by high quality and broad coverage. It can reflect tourists’ expectations of local specialty products, so we use it as a data source. In this study, we successfully crawled 9428 user reviews from Ctrip using a web crawler code written in Python. During the data cleaning process, we identified and removed duplicate reviews, ultimately retaining 7964 valid reviews. These reviews cover various famous scenic spots in Qiandongnan, such as Xijiang Qianhu Miao Village, Zhaoxi Dong Village, Zhenyuan Ancient Town, and Xiasi Ancient Town.

After pre-processing the reviews, we employed keyword matching and synonym expansion techniques in NLP to filter out reviews related to cultural tourism products. Analyzing these reviews yielded the following conclusions: Qiandongnan is favored by tourists for its rich ethnic culture and delicious food. However, there are also serious issues of commercialization and homogenization of cultural tourism products, which lack distinctive local characteristics and cultural connotations. Based on this, we summarized consumers’ requirements for cultural tourism products, which are distinctive local characteristics and food-based themes.

**Figure 5:**
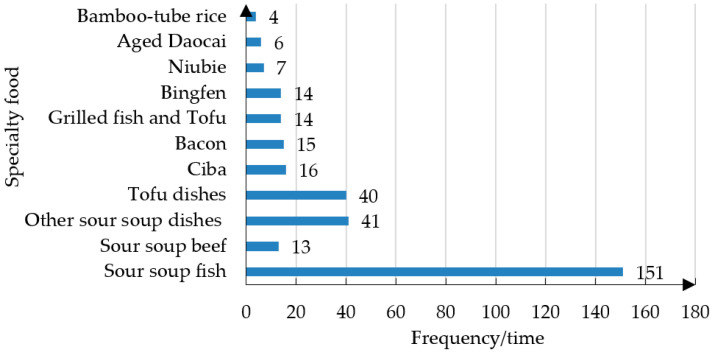
The statistics of specialty foods in the Qiandongnan.

In addition, we analyzed the frequency of food items mentioned in the reviews, and the results are shown in Figure 5. We found that RRS foods (including sour soup fish, sour soup beef, and other sour soup foods) accounted for the highest proportion, with sour soup fish being mentioned 151 times. This result further confirms tourists’ fondness for RSS.

#### 4.1.2. Consumer Preference Analysis Based on LDA

JD.com and Taobao are representative platforms of B2C and C2C models in China. These platforms gather authentic feedbacks from consumers with diverse regional and demographic backgrounds, ensuring high authenticity and diversity in the data. Extracting consumer preferences for Guizhou RSS packaging from these sources provides highly representative and valuable insights. Considering the dynamic nature of consumer requirements, we excluded data prior to March 2018. Instead, we focused on collecting all reviews from March 2018 to May 2024, ensuring that our analysis reflects current trends. In this study, we collected 4516 unique RSS reviews. After a series of pre-processing operations on these reviews, including removing symbols and stop words, Chinese word segmentation and integration, we conducted topic mining.

**Figure 6:**
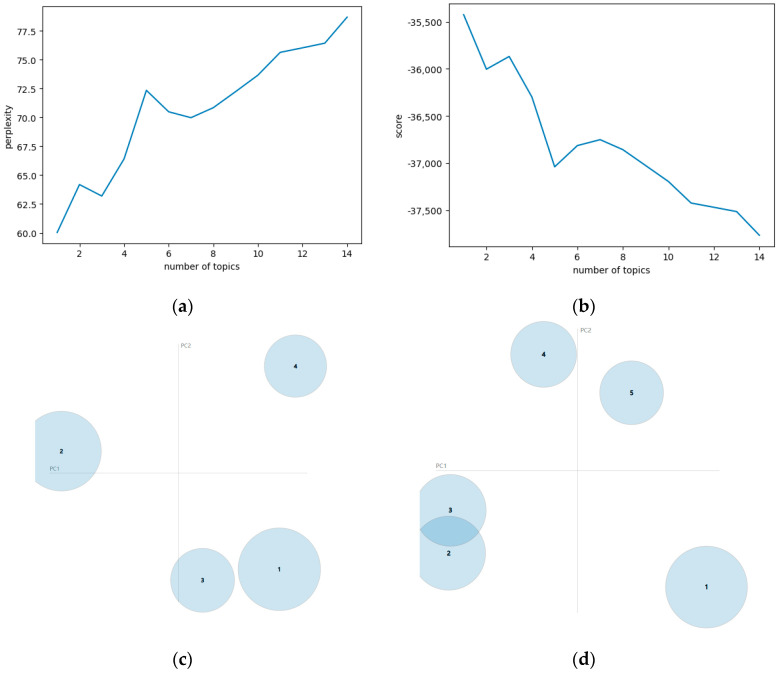
Topic number selection for the LDA model. (**a**) Perplexity; (**b**) coherence; (**c**) distribution for four topics; (**d**) distribution for five topics.

In the application of LDA, to determine the optimal number of topics, we calculated and compared the perplexity and coherence under different numbers of topics, and the results are shown in Figure 6a,b. When the number of topics is 4 or 5, it shows good continuity and interpretability. Furthermore, we plotted the topic distribution for 4 and 5 topics, as shown in Figure 6c,d, respectively. From Figure 6d, it can be observed that there is an overlap between Topic 3 and Topic 2. Therefore, the number of topics is determined to be 4.

**Figure 7:**
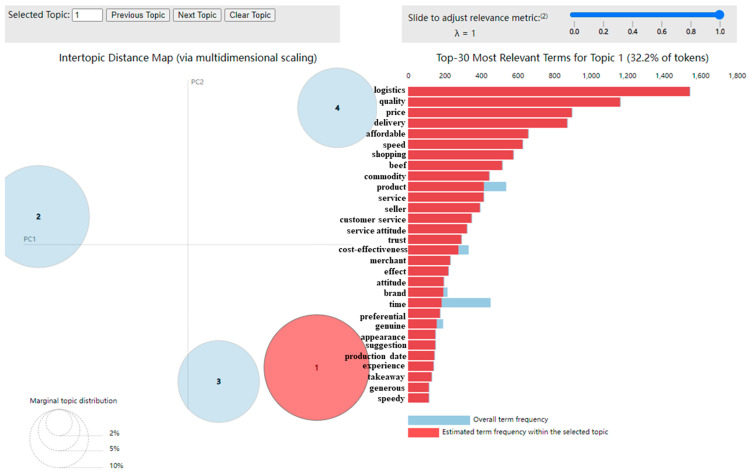
Topics and their weight distribution.

We built the LDA model using the Gensim library in Python, and the model parameter settings are shown in Table 3. Figure 7 presents the mining results, including four topics and their weight distribution. For a more intuitive presentation, we provide the top 25 feature words with the highest weights under each topic in Table 4.

According to the results of topic mining, we can summarize consumers’ focus into four topics: shopping experience, food quality, packaging and cooking, and specialty food. To further analyze packaging, we filtered out and organized the reviews in Topic 3. Table 5 shows some of the results.

Based on the above analysis, we summarized consumers’ preferences for RSS packaging, which include optimizing usage instructions, clearly labeling information, improving bottle quality, optimizing packaging design, etc. These suggestions provide important references for the subsequent packaging design of RSS.

#### 4.1.3. Consumer Semantic Space

**Figure 8:**
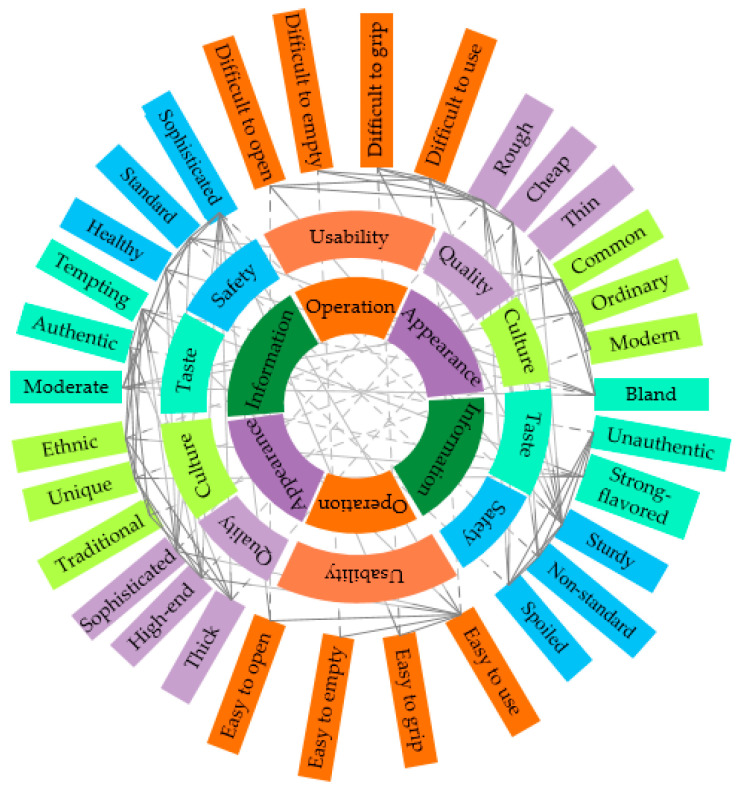
The semantic network of Kansei words. Solid lines indicate associations; dashed lines indicate a pair of Kansei words.

On the basis of analyzing the dual requirements of consumers’ expectations for cultural tourism products and their preferences for RSS, we integrated them into three categories: informational, operational, and appearance-related. We further divided them into five perceptual dimensions: usage, culture, taste, quality, and safety. Each dimension contains 6–8 Kansei words. To visualize the intrinsic connection and structure among the 32 collected Kansei words, we constructed a semantic network, as shown in Figure 8.

In the selection of key nodes, i.e., key Kansei words, we used degree centrality as the indicator. Degree centrality refers to the number of connections between Kansei words, and the results are shown in Table 6. We selected the word pairs with the highest values from each dimension (shown in bold) as key Kansei words.

Specifically, in the usage dimension, we selected “easy to use—difficult to use” to evaluate the convenience of opening, pouring, and grasping the container. For simplicity, we replaced it with “easy—difficulty” in the following text. In the quality dimension, “sophisticated—rough” was chosen to reflect the quality of food packaging. In the culture dimension, “ethnic—common” was selected to assess the cultural connotation, as it captures the essence of whether the packaging design reflects the unique cultural characteristics of the Miao ethnic group or appears more common. For the taste dimension, we used “tempting—bland” to evaluate the evocation of the consumer’s taste imagination. In the safety dimension, “sturdy—deformable” was selected to evaluate the psychological feelings brought to consumers by the packaging in terms of pressure resistance, shock resistance, and leak prevention.

### 4.2. Product Attribute Space Construction

#### 4.2.1. Determination of Design Elements

Considering the liquid characteristics of RSS and consumer reviews, we chose to continue using bottles. To optimize the appearance of the bottle, we collected various types of bottles through market research. A morphological analysis was used to decompose the bottle into five independent design elements: body material, cap type, shoulder shape, body shape, and surface decoration. These five components were further divided into 13 specific design levels, as shown in detail in Table 7.

Regarding the consideration of packaging materials, the high acidity of RSS may corrode metals, so we excluded it. In addition, based on the consumer requirement analysis conducted in the previous section, we know that product label information (e.g., product name, ingredients, license number, production date, shelf life, place of origin, net content, usage instructions, etc.) and gift boxes are essential in RSS packaging design. Therefore, we treated them as necessary elements and directly added them to the design scheme in the detailed design phase.

#### 4.2.2. Sample Construction

Using the full-profile method for experimental design would result in $C_{3}^{1}*C_{2}^{1}*C_{2}^{1}*C_{4}^{1}*C_{2}^{1}=96$ combinations of samples. Such a large number of samples would not only impose a cognitive burden on the subjects but could also lead to attention fatigue, thereby affecting the accuracy of the experimental results. To mitigate this issue, we adopted the orthogonal method for experimental design. The orthogonal method is a fractional factorial design that selects a subset of the full factorial design while maintaining a balanced and representative sample of the design space. This method is commonly used with sample sizes of 8, 16, and 36 for different scales. Our study incorporated five design factors, each with 2–4 levels, resulting in a relatively small design space. After analyzing, we chose 16 sample sizes, and their encoding results are shown in Table 8.

**Figure 9:**
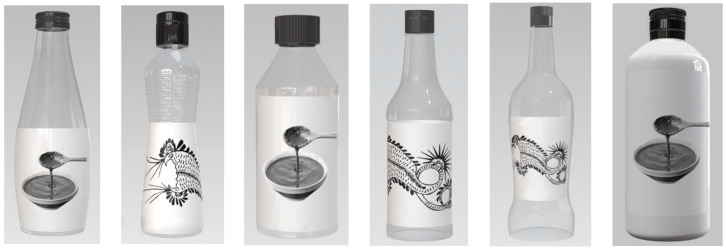
Display of some samples. From left to right, they are samples 4, 5, 8, 10, 14, and 15.

Based on the coding results in Table 8, we used Photoshop to create images for the 16 samples, and Figure 9 shows some of them. Compared to textual descriptions, images allow subjects to intuitively feel the perception conveyed by the combination of design elements, which is why sample images were required. In addition, to eliminate the interference of other variables and maintain design consistency, we used white bottle labels and black caps for all sample images.

### 4.3. Questionnaire Survey

**Figure 10:**
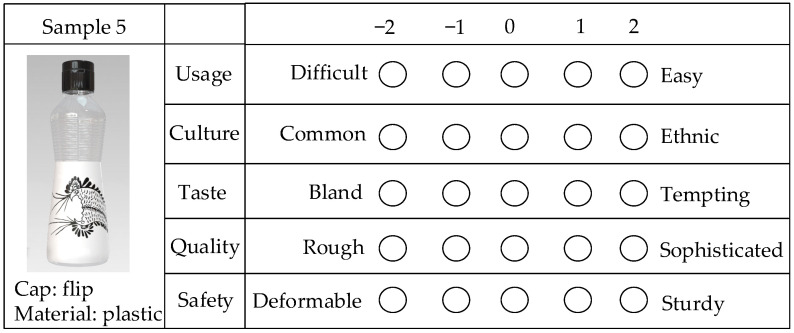
One of the packaging evaluation questionnaires. The first column contains the sample information, the second column lists the evaluation dimensions, and the third column presents the corresponding Kansei word pairs. Subjects rate the packaging by marking circles on the SD scales.

We designed the questionnaire using structured closed-ended questions, as shown in Figure 10. The SD scale used in the questionnaire ranges from −2 to 2, where negative values represent a tendency towards the Kansei word on the left, positive values lean towards the one on the right, and zero indicates neutrality. Considering that images alone might not convey detailed information such as the material and cap of the samples, we supplemented them with textual descriptions to ensure comprehensive expression.

To ensure the validity of the questionnaire responses, we focused on two main groups of participants: local consumers from Kaili and surrounding areas in Guizhou Province, and tourists from other provinces visiting the region. Kaili and its surrounding areas are known for their production and consumption of RSS, making it an ideal location to find qualified local participants. Furthermore, as RSS is a popular souvenir among tourists, we also sought to include their perspectives on RSS packaging. We screened both groups of participants by asking about their prior experience with RSS to confirm their familiarity with the product.

The questionnaire was created on the Wenjuanxing platform, which generates a QR code for easy distribution. We employed both online and offline channels to invite participants. Online, we shared the questionnaire through social media platforms such as WeChat and QQ. Offline, we approached potential participants in Kaili, including local residents and tourists, allowing them to scan the QR code directly. Our survey did not disclose any personal information of the subjects and complied with relevant regulations.

After conducting the survey, we collected a total of 120 valid questionnaires. By organizing the survey results and calculating the means, we obtained the Kansei matrix. Furthermore, using Equation (5), we calculated that the Cronbach’s α coefficient was 0.8273. According to Table 2, 0.8273 > 0.8 indicates that the Kansei matrix has very high reliability and can be used for the next stage of research.

### 4.4. Mapping Model Construction

This study used conjoint analysis to analyze the 16 samples and the Kansei matrix. We wrote data-processing code using Python to calculate importance values and utility values, as shown in Table 9 and Table 10.

Table 9 and Table 10 show that in the usage dimensions, the cap is the most important factor (48.07%), followed by the shoulder (23.66%), while the body (12.02%) and image (11.45%) have less impact. Specifically, the flip cap is easy to use (1), while the screw cap is difficulty of use (−1). The flat shoulder is easy to use (0.429), while the sloped shoulder is difficult to use (−0.556).

In the culture dimension, the image is the dominant factor (42.71%), followed by the cap (27.53%), while the body (14.95%) and material (11.96%) have less impact. The cultural image has a high utility value of 1.714, showing its importance in cultural communication. The flip cap (1.571) performs better than the screw cap (1.111) in the culture dimension.

In the quality dimension, the material is the most important factor (58.11%), followed by the body (24.21%). Ceramic and glass tend towards refinement, with utility values of 1.4 and 1.33, respectively, while plastic appears relatively simple (−1). Convex, cone, and flat all lean towards sophisticated.

In the taste dimension, the material is the most important factor (41.94%), followed by the cap (27.02%) and then the body (18.50%). Glass performs best in stimulating taste experience (1.5), followed by ceramic (1), while plastic performs the worst (−0.2). The high score of glass in taste perception may be attributed to its aesthetic appeal and the psychological associations consumers have with the material. On the one hand, glass scores high in the quality dimension (1.4), enhancing the aesthetic perception of the product. On the other hand, the transparency of glass allows consumers to see the RSS inside, which heightens anticipation and positively influences taste perception. In terms of cap design, the flip cap stimulates taste experience more (1.429).

In the safety dimension, the material is the most influential factor (60.28%), while the shoulder (7.18%) and image (3.76%) have the least impact. Ceramic is the strongest in conveying a sense of safety (1.8), followed by glass (1.5), and plastic performs the worst (−1).

An unexpected finding is the significant influence of bottle cap on the culture dimension, indicating that even functional design elements can play an important role in cultural communication. This emphasizes the need to comprehensively consider the multidimensional impact of each element in packaging design. The conjoint analysis results have high reliability and validity, verifying intuitive expectations (such as the advantages of glass and ceramic materials in quality and safety) and revealing non-intuitive but important insights. These findings provide clear guidance and a solid basis for optimizing the packaging design of RSS, helping designers better understand and meet consumer requirements.

### 4.5. RSS Packaging Design

In the process of promoting local specialty foods, packaging needs to resonate with consumers, showing the characteristics of the food itself and conveying the cultural connotations behind it. This study uses semiotics and Kansei engineering to guide the packaging design of RSS, aiming to improve the adaptability of batik culture and packaging design. The design process includes five key steps: requirement positioning, element extraction, functional planning, detailed design, and scheme evaluation.

In the requirement positioning stage, based on the design guidance strategies obtained in Section 4.4, we developed the design levels for RSS packaging from five dimensions. Specifically, for usage, we adopted a flip cap and slanted shoulder; for culture, we used batik patterns; considering the conveyance of taste sensation, we excluded opaque ceramic materials to avoid the product giving a bland impression; for safety, we eliminated plastic; for quality, we employed batik pattern and glass to convey a sense of sophistication. Ultimately, we determined glass (*x*_1-1_), a flip cap (*x*_2-1_), a slanted shoulder (*x*_3-2_), a cone shape (*x*_4-3_), and cultural images (*x*_5-2_).

**Figure 11:**
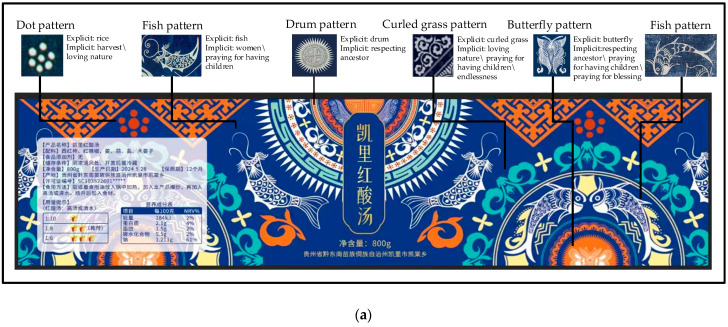

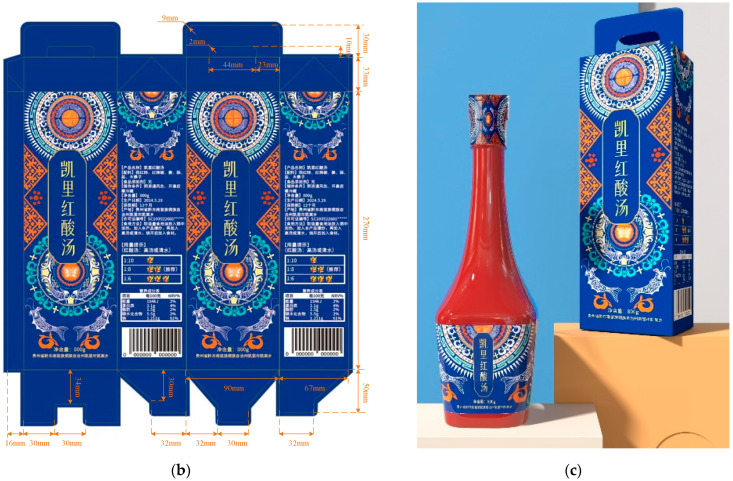
RSS packaging design scheme. (**a**) Bottle label design; (**b**) packaging box design; (**c**) renderings. Our design scheme is just for display. For some sensitive or inappropriate public information, we have used asterisks (*) or the number 0 as substitutes.

In the element extraction stage, based on the previously constructed batik knowledge graph [39], we selected patterns like dots, fish, drum, curled grass, and butterfly. When choosing patterns, it is crucial to consider cultural taboos and avoid inauspicious meanings, such as those related to funerals. For example, the horseshoe pattern, often used to cover the body of a deceased male, is inappropriate for product packaging. The semantic analysis reveals that these patterns carry meanings such as loving nature, the harvest, and praying for a blessing, with the detailed semantics shown in Figure 11a. Notably, in Miao culture, certain pattern combinations may have special meanings, like the fish and bird combination representing the love between husband and wife. Therefore, when selecting patterns, we should fully understand their meanings to ensure that the design conforms to cultural connotations and meets modern aesthetic requirements.

In the context dimension-oriented functional planning stage, we constructed three main scenarios: purchasing from supermarkets or online platforms, as tourist souvenirs, and as gifts. For these scenarios, we planned the following contents: (i) in terms of color design, we primarily use blue and white from batik, incorporating auxiliary colors such as orange, cyan, and yellow. This combination not only enhances recognition and ethnic characteristics but also forms a strong visual attraction. (ii) In terms of information design, we clearly label food information, including production date, shelf life, consumption methods, and dosage instructions, among others. (iii) We develop a gift box that is consistent with the product style, taking into account both practicality and aesthetics, suitable as tourist souvenirs or gifts.

In the syntactic dimension-oriented detailed design stage, we fully considered the Miao people’s aesthetic characteristics of symmetry and fullness. In the overall layout, we adopted a highly symmetrical circular layout, highlighting the product name “凯里酸汤鱼”(Kaili RSS) in the center, surrounded by various Miao pattern elements. In the combination of symbols, various patterns are organically integrated, maintaining the uniqueness of each pattern while forming a harmonious and unified visual effect. Regarding proportions, the ratio of decorative elements to informational content is about 3:1, both highlighting cultural characteristics and ensuring the conveyance of information. The final design scheme is shown in Figure 11.

In the design evaluation stage, we conducted a comparative analysis between our scheme (new packaging) and the traditional packaging (old packaging) shown in Figure 1c. A total of 120 subjects evaluated the designs using a SD scale ranging from −2 to 2, and the results are summarized in Table 11.

The new packaging significantly outperforms the old packaging across all evaluated dimensions (all *p* < 0.001), with large effect sizes (r > 0.85) consistently observed. The dimensions of culture (Mean = 1.90, Median = 2.00) and overall satisfaction (Mean = 1.85, Median = 2.00) show particularly strong improvements, with minimal variability (IQR = 0.00) in consumer ratings. The old packaging design, in contrast, received consistently negative evaluations across all dimensions (Mean ranges from −1.75 to −1.50, Median = −2.00).

The most significant change, apart from appearance, was the material change from plastic to glass. While plastic bottles have advantages in terms of cost due to low raw material costs and simple production processes, glass bottles align better with sustainable practices and environmental considerations. Glass is easily recyclable, has low processing costs, and has a relatively lower environmental impact compared to plastic.

**Figure 12:**
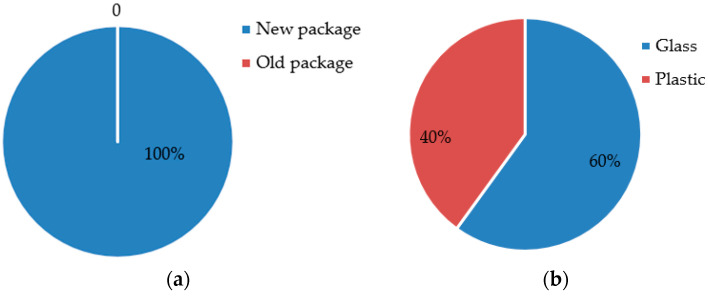
Packaging comparison results. (**a**) The choice between new and old packaging; (**b**) the choice between glass and plastic material.

To understand consumers’ purchasing intentions and attitudes towards packaging materials, we conducted another survey. The questionnaire was designed with two questions: (i) If the price and material are the same, will subjects choose the new or the old packaging? (ii) With the new packaging, given that glass is more expensive than plastic, what material will the subjects choose? It should be noted that this survey is only to compare the design and material, and the soup inside is exactly the same. After distributing the questionnaires to 120 subjects, we obtained the results shown in Figure 12.

As shown in Figure 12a, 100% chose the new packaging, demonstrating the effectiveness and feasibility of our design. More encouragingly, Figure 12b reveals that 60% of participants were willing to choose glass packaging even with a price increase. To further examine the statistical significance of consumers’ material preference, we conducted a chi-square goodness-of-fit test. Among 120 participants, the observed frequencies were 72 for glass and 48 for plastic packaging. Under the null hypothesis of no material preference, the expected frequencies were set at 60 for each material. The test results yielded a chi-square value χ2 = 4.8 (exceeding the critical value of 3.84 at the 0.05 significance level), *p* = 0.028 < 0.05, indicating a significant preference for packaging materials. This preference demonstrates consumers’ strong tendency toward glass materials, reflecting their emphasis on quality and environmental considerations.

Through a series of evaluations and comparisons, this study provides data support for the packaging design of RSS, which has significant theoretical and practical implications for guiding future food packaging design.

## 5. Conclusions

Kaili RSS, a local specialty food favored by Miao people and tourists, faces market limitations due to packaging that inadequately engages consumer attention. This study developed an integrated research framework combining web crawling, NLP, LDA, Kansei engineering, and semiotics. By constructing a mapping model between consumer perceptual requirements and packaging design elements, we incorporated batik patterns into food packaging, developing a solution that combines both practicality and aesthetics.

Our research covers the entire process from consumer requirement analysis to packaging design implementation, offering both practical solutions for Kaili RSS and methodological insights for other traditional food packaging. Despite these contributions, several limitations should be noted. Primary limitations include the exclusive focus on online consumer data and the omission of design elements such as colors, typography, and font sizes in attracting consumer attention. In future research, we will address these limitations to enhance the expressiveness and appeal of packaging design, aiming to strengthen local specialty foods’ competitiveness in both domestic and international markets.

## Figures and Tables

**Table 1:** Summary of cultural applications in food packaging design.

Reference	Case	Design Scheme	Design Method	Problem	Strategy
Zhao [9]	No	No	No	Poor packaging Low value added No innovation	Local specialty branding Consumer-based positioning
Liu [10]	No	No	No	Poor cultural adaptation No innovation Poor eco-awareness Design–market disconnect	Cultural exploration Integrate cultural elements Natural material application Focus on market and consumers
Liu et al. [11]	Apple	No	No	Poor design awareness Lack of cultural elements Poor eco-awareness Poor information delivery	Enhance brand consciousness Integrate cultural elements Promote eco-packaging Optimize design components
Wu and Chen [12]	Tea\ milk\ noodles	Pre-existing	No	Poor cultural understanding Appearance over culture No innovation	In-depth cultural exploration Traditional–modern integration
Ours	RSS	Original	Kansei engineering\Semiotics	Poor packaging Poor cultural adaptation Neglects the consumer Lack of design methodology	Enhance design awareness Explore batik culture Semiotic for cultural adaptation Kansei engineering and reviews for consumer requirements

**Table 2:** Value range of Cronbach’s α coefficient.

α	0 < α < 0.6	0.6 < α < 0.7	0.7 < α < 0.8	0.8 < α < 0.9	0.9 < α < 1
Internal consistency	Unacceptable	To be examined	Acceptable	Good	Excellent

**Table 3:** Training parameter setting.

Parameters	Setup	Meaning
n_components	4	Number of topics
max_iter	50	Max number of iterations
learning_method	batch	Learning method
learning_offset	50	Learning offset
random_state	0	Random seed

**Table 4:** Review content topic distribution.

Topic	Summary	Feature Words
Topic 1	Shopping experience	Logistics\quality\price\delivery\affordable\speed\shopping\beef\commodity\product\service\seller\customer service\service attitude\trust\cost-effectiveness\merchant\effect\ attitude\brand\time\preferential\genuine\appearance\suggestion\production date\experience\takeaway\generous\speedy
Topic 2	Product quality	Authentic\taste\evaluation\sour\try\brand\merchant\local\comment\feeling\activity\ brand\beef\try\great\habit\product\hometown\real thing\red soup\aroma\personal\ addictive\cheap\problem\colleague\physical store\variety\word count\imagination
Topic 3	Packaging and cooking	Taste\feeling\some\quantity\quality\basic ingredients\bottle\appearance\additives\ spicy\pepper\color\flavor\packaging\child\add\cooking\sourness\appetite\ ingredients\soup material\add\red sour\calories\seasoning\production date\ ingredients\tomato\appetite\go home
Topic 4	Specialty food	hot pot\taste\friend\seasoning\delicious\soup base\specialty\tourism\order\flavor\ noodles\vegetables\pig feet\sour fish\food\specialty\rice noodles\delicacy\quality\ Miao\tomato\sourness\beef\tongue\fish\appetite\basic\consumption\vegetable\tofu

**Table 5:** Packaging requirements extraction.

Users	Reviews	Requirements
Hua***8	Followed the label, half a bottle for 4 people. Still way too sour and spicy. Even sugar didn’t help.	Optimizing usage instructions
j***5	Review after eating. There’s an expiration date, but no make date.	Clearly labeling information
j***0	The bottle allows you to pour out as much as you need, very convenient.	Easy to pour
j***b	Bottle quality needs improving. Twisting the cap warps the whole thing and it’s still not open.	Easy to open
e***8	One of the bottles was broken. Please improve packaging.	Improving bottle quality
j***g	One of the bottles was deformed.	Improving bottle quality
k***8	No gift box, just two bottles in a crap box. How am I supposed to gift this? Zero attention to detail.	Optimizing packaging design
na***a	These water bottles look like cheap knockoffs. Couldn’t they change the packaging.	Optimizing packaging design

**Table 6:** Kansei word pairs and degree centrality.

Classification	Dimension	Kansei Word Pairs	Degree Centrality
Operation	Usage	**Easy to use–Difficult to use**	13
		Easy to grip–Difficult to grip	5
		Easy to empty–Difficult to empty	3
		Easy to open–Difficult to open	5
Appearance	Quality	**Sophisticated–Rough**	15
		High-end–Cheap	11
		Thick–Thin	6
	Culture	**Ethnic**–**Common**	11
		Unique–Ordinary	10
		Traditional–Modern	6
Information	Taste	**Tempting–Bland**	11
		Authentic–Unauthentic	9
		Moderate–Strong-flavored	9
	Safety	**Sturdy–Deformable**	17
		Standard–Non-standard	11
		Healthy–Spoiled	11

Note: The bolded ones are the selected core Kansei word pairs.

**Table 7:** Design elements.

No.	Design Element	Design Level			
1	Material (*x*_1_)	Glass(*x*_1-1_)	Plastic(*x*_1-2_)	Ceramic(*x*_1-3_)	
2	Cap(*x*_2_)	Flip(*x*_2-1_)	Screw(*x*_2-2_)		
		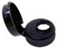	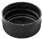		
3	Shoulder (*x*_3_)	Flat (*x*_3-1_)	Slanted (*x*_3-2_)		
		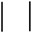	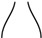		
4	Body (*x*_4_)	Concave (*x*_4-1_)	Convex (*x*_4-2_)	Cone (*x*_4-3_)	Flat (*x*_4-4_)
		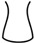	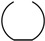	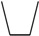	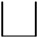
5	Image (*x*_5_)	Product (*x*_5-1_)	Cultural (*x*_5-2_)		
		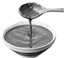	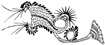		

**Table 8:** Sample coding.

No.	*x_1_*			*x_2_*		*x_3_*		*x_4_*				*x_5_*	
	*x*_1-1_	*x*_1-2_	*x*_1-3_	*x*_2-1_	*x*_2-2_	*x*_3-1_	*x*_3-2_	*x*_4-1_	*x*_4-2_	*x*_4-3_	*x*_4-4_	*x*_5-1_	*x*_5-2_
1	1	0	0	1	0	1	0	1	0	0	0	1	0
2	1	0	0	1	0	1	0	0	1	0	0	0	1
3	1	0	0	0	1	0	1	0	0	1	0	1	0
4	1	0	0	0	1	0	1	0	1	0	0	1	0
5	0	1	0	1	0	0	1	1	0	0	0	0	1
6	0	1	0	1	0	1	0	0	1	0	0	1	0
7	0	1	0	0	1	1	0	0	0	1	0	0	1
8	0	1	0	0	1	0	1	0	0	0	1	1	0
9	0	0	1	1	0	1	0	0	0	1	0	1	0
10	0	0	1	1	0	1	0	0	0	0	1	0	1
11	0	0	1	0	1	0	1	1	0	0	0	1	0
12	0	0	1	0	1	0	1	0	1	0	0	0	1
13	1	0	0	1	0	0	1	0	0	1	0	0	1
14	0	1	0	0	1	0	1	1	0	0	0	0	1
15	0	0	1	0	1	0	1	0	0	0	1	1	0
16	1	0	0	0	1	1	0	0	0	0	1	1	0

**Table 9:** Importance values.

Dimension	Packaging Material/%	Cap/%	Shoulder/%	Body/%	Image/%
Usage	4.81	48.07	23.66	12.02	11.45
Culture	11.96	27.53	2.85	14.95	42.71
Taste	41.94	27.02	8.22	18.5	4.31
Quality	58.11	9.99	3.84	24.21	3.84
Safety	60.28	12.64	7.18	16.15	3.76

**Table 10:** Utility values.

Design Element	Level	Usage	Culture	Taste	Quality	Safety
Packaging material	Glass	0	1.333	1.5	1.333	1.5
	Plastic	−0.2	1.2	−0.2	−1	−1
	Ceramic	−0.2	1.4	1	1.4	1.8
Cap	Flip	1	1.571	1.429	0.857	1.143
	Screw	−1	1.111	0.333	0.444	0.556
Shoulder	Flat	0.429	1.286	1	0.7143	1
	Slanted	−0.556	1.333	0.667	0.556	0.667
Body	Concave	0	1.25	0.75	0	0.25
	Convex	0	1.5	1.25	1	1
	Cone	0	1.25	0.75	0.75	1
	Flat	−0.5	1.25	0.5	0.75	1
Image	Product	−0.333	1	0.889	0.556	0.889
	Culture	0.143	1.714	0.714	0.714	0.714

**Table 11:** Mann–Whitney U test results for packaging design evaluation.

	Mean		Median		IQR		r	*p*
	New	Old	New	Old	New	Old		
Usage	1.85	−1.65	2	−2	0	1	0.863	<0.001
Culture	1.9	−1.55	2	−2	0	1	0.864	<0.001
Taste	1.55	−1.5	2	−2	1	1	0.858	<0.001
Quality	1.80	−1.75	2	−2	0	0	0.861	<0.001
Safety	1.45	−1.65	1	1	−2	1	0.860	<0.001
Satisfaction	1.85	−1.65	2	−2	0	1	0.864	<0.001

## Data Availability

The original contributions presented in the study are included in the article. Further inquiries can be directed to the corresponding author.
